# Patient-reported outcomes of baricitinib in patients with rheumatoid arthritis and no or limited prior disease-modifying antirheumatic drug treatment

**DOI:** 10.1186/s13075-017-1410-1

**Published:** 2017-09-18

**Authors:** Michael Schiff, Tsutomu Takeuchi, Roy Fleischmann, Carol L. Gaich, Amy M. DeLozier, Douglas Schlichting, Wen-Ling Kuo, Ji-Eon Won, Tara Carmack, Terence Rooney, Patrick Durez, Saeed Shaikh, Rodolfo Pardo Hidalgo, Ronald van Vollenhoven, Cristiano A. F. Zerbini

**Affiliations:** 1 University of Colorado School of Medicine, Denver, CO 80045 USA; 2 Keio University, Tokyo, Japan; 3 Metroplex Clinical Research Center, University of Texas Southwestern Medical Center, Dallas, TX 75231 USA; 4 Eli Lilly and Company, Indianapolis, IN 46285 USA; 5 Eli Lilly and Company, Seoul, Republic of Korea; 6 Service et Pôle de Rhumatologie, Cliniques Universitaires Saint-Luc, Brussels, Belgium; 7 Institut de Recherche Expérimentale et Clinique, Université catholique de Louvain, Rheumatology, Brussels, Belgium; 8 McMaster University, Hamilton, ON Canada; 9 CER, San Juan, Argentina; 10 Amsterdam Rheumatology and Immunology Center ARC, Amsterdam, The Netherlands; 11 Centro Paulista de Investigação Clinica, São Paulo, Brazil

**Keywords:** Baricitinib, PRO, JAK inhibitor, RA, Rheumatoid, tsDMARD, Health-related quality of life, health status indicators, HAQ-DI

## Abstract

**Background:**

This study evaluates patient-reported outcomes (PROs) in a double-blind, phase III study of baricitinib as monotherapy or combined with methotrexate (MTX) in patients with active rheumatoid arthritis (RA) with no or minimal prior conventional synthetic disease-modifying antirheumatic drugs (DMARDs) and naïve to biological DMARDs.

**Methods:**

Patients were randomized 4:3:4 to MTX administered once weekly (*N* = 210), baricitinib monotherapy (4 mg once daily (QD), *N* = 159), or combination of baricitinib (4 mg QD) and MTX (baricitinib + MTX, *N* = 215). PROs included the Patient’s Global Assessment of Disease Activity (PtGA), patient's assessment of pain, Health Assessment Questionnaire-Disability Index (HAQ-DI), Functional Assessment of Chronic Illness Therapy-Fatigue (FACIT-F), duration of morning joint stiffness (MJS), worst joint pain, worst tiredness, Work Productivity and Activity Impairment-Rheumatoid Arthritis (WPAI-RA), Short Form 36 version 2, Acute (SF-36); and EuroQol 5-Dimensions (EQ-5D) Health State Profile. Comparisons were assessed with analysis of covariance (ANCOVA) and logistic regression models.

**Results:**

Compared to MTX, patients in both baricitinib groups reported greater improvement (*p* ≤ 0.01) in HAQ-DI, PtGA, pain, fatigue, worst join pain, SF-36 physical component score, and EQ-5D at weeks 24 and 52. For the SF-36 mental component score, patients in both baricitinib groups reported statistically significant improvements (*p* ≤ 0.01) at week 52 compared to MTX-treated patients. Statistically significant improvements (*p* ≤ 0.05) were observed with the WPAI-RA for the baricitinib groups vs. MTX at week 24 and for the WPAI-RA daily activity and work productivity measures for baricitinib + MTX at week 52.

**Conclusions:**

In this study, baricitinib alone or in combination with MTX, when used as initial therapy, resulted in significant improvement compared to MTX in the majority of the pre-specified PRO measures.

**Trial Registration:**

ClinicalTrials.gov, NCT01711359. Registered on 18 October 2012.

## Background

Patients with rheumatoid arthritis (RA) experience progressive and significant restrictions in daily living and frequently report pain, fatigue, sleep disturbances, and functional impairment in work and leisure-time physical activities [1–3]. Patient-reported symptoms are generally present early in the disease, and, when present, can be a significant burden on patients’ quality of life. The American College of Rheumatology (ACR) established a core set of measures to assess disease activity in clinical trials, which includes several patient-reported outcomes (PROs) associated with RA [4]. Additional PROs for use in clinical trials have been suggested by Outcome Measures in Rheumatology Clinical Trials (OMERACT) [5]. The ACR and the European League Against Rheumatism (EULAR) have recognized PROs as important factors in the assessment of patient disease activity and have recommended the evaluation of PROs in daily clinical practice when considering response to therapy [6, 7]. Regulatory authorities have recommended that PROs be used as additional measures of effectiveness in clinical trials of RA [8].

Treatment recommendations for RA highlight the importance of early diagnosis and of intensive treatment strategies, with the target of remission or lowest possible disease activity [9]. The treatment regimen for RA suggested by the ACR and EULAR includes initial conventional synthetic disease-modifying anti-rheumatic drugs (csDMARDs), especially methotrexate (MTX); if not effective, combining csDMARDs with biological DMARDs (bDMARDs), or oral targeted synthetic DMARDs (tsDMARDs) that target the intracellular Janus kinase (JAK) pathways, are recommended [6, 7]. This early intensive disease management has been shown to mitigate joint damage and inflammation, to avoid disability from RA, and to improve patient health-related quality of life [10–13].

It has been advocated that the treatment of RA should be a shared decision-making process between the patient and the physician [9]. Together, they must address questions of significance to the patients, including the potential improvement of PROs [9]. A relevant question to both patients and physicians is whether bDMARDs or tsDMARDs require co-administration of methotrexate (MTX), which itself is associated with potential adverse effects that may affect patient function (fatigue, nausea, etc.) [14, 15].

Baricitinib is a selective JAK1 and JAK2 inhibitor that was recently approved for the treatment of moderately to severely active RA in adults in the European Union and is under development for RA in other regions. Baricitinib interferes with pathways that are believed to be important in the pathogenesis of RA. RA-BEGIN was a phase III study (NCT01711359) conducted in patients with active RA who were naïve to csDMARDs (no or limited exposure to MTX) or bDMARDs. Baricitinib alone or in combination with MTX demonstrated superior clinical efficacy with acceptable safety compared to MTX as the initial therapy for patients with active RA [1]. In the present analysis of the RA-BEGIN study, we report the effects of baricitinib, administered as monotherapy or in combination with MTX compared to MTX monotherapy, on the PRO measures.

## Methods

### Patients

Full details regarding the primary efficacy and safety outcomes of this study have been reported previously [1]. In summary, patients were ≥ 18 years old with moderately to severely active RA (≥6/68 tender and ≥ 6/66 swollen joints; high-sensitivity C-reactive protein ≥ 3.6 mg/L (upper limit of normal 3.0 mg/L); seropositive for rheumatoid factor or anti-citrullinated peptide antibodies) and naïve (or had no more than three prior doses of MTX) to DMARDs.

### Study protocol and oversight

RA-BEGIN was a randomized, 52-week, double-blind, active comparator-controlled study conducted in 18 countries. Patients were randomized 4:3:4 to receive oral MTX monotherapy (administered orally once weekly), baricitinib monotherapy (4 mg once daily (QD)), or the combination of baricitinib (4 mg QD) and MTX (baricitinib + MTX). Methotrexate was initiated at 10 mg/week and, if tolerated, increased to 20 mg/week by week 8. A lower dose of MTX was available for patients in whom a lower dose was clinically indicated or required by national guidelines (initial dose of 7.5 mg and a maximum dose of 12.5 mg). Rescue treatment (baricitinib + MTX) was available, beginning at week 24, for those patients whose tender and swollen joint counts did not improve by ≥ 20% from baseline. The study was conducted in accordance with ethical principles of the Declaration of Helsinki and Good Clinical Practice guidelines and was approved by each center’s institutional review board or ethics committee. All patients provided written informed consent.

### Patient-reported outcomes

Pre-specified secondary PROs were included in the study. The Patient’s Global Assessment of Disease Activity (PtGA) and the patient’s assessment of pain were evaluated using visual analog scales (VAS) of 0–100 mm. Physical function was assessed by the Health Assessment Questionnaire-Disability Index (HAQ-DI) [2, 3]; scores range from 0 to 3, with lower scores reflecting better physical function and less disability. An HAQ-DI score of ≤ 0.25 is considered the normative value and an improvement of ≥ 0.22 has been shown to be the minimum clinically important difference (MCID) [16, 17]. Fatigue was assessed by the Functional Assessment of Chronic Illness Therapy-Fatigue (FACIT-F) scale; scores range from 0 to 52, with higher scores representing less fatigue; in this study a value of 3.56 was used for the MCID [18–20]. Duration of morning joint stiffness (MJS) was reported by the patient as the length of time in minutes that MJS lasted on the day prior to each study visit. The patients’ assessments of the worst joint pain and the worst tiredness over the past 24 hours were measured using novel Worst Joint Pain and Worst Tiredness Numeric Rating Scales (NRS). Scores for both the NRSs range from 0 (no joint pain/tiredness) to 10 (“as bad as you can imagine”).

Health-related quality of life (HRQOL) was evaluated using the Medical Outcomes Study (MOS) Short Form-36 (SF-36; version 2, Acute) [21, 22], which assesses eight domains scored from 0 to 100 that can be aggregated into physical and mental component scores (PCS, MCS) and compared to values from normal individuals. An MCID change of 5 was used to assess the clinical relevance of changes in SF-36 component scores [23, 24]. The EuroQol 5-Dimensions (EQ-5D) Health State Profile was also used to assess HRQOL. The EQ-5D consists of two components: a descriptive system of the respondent’s health and a rating of their current state (0 − 100 mm VAS) [25]. The UK and US scoring algorithms provide an index score using the UK or US population weighting normalized to that population [25–27].

The Work Productivity and Activity Impairment-Rheumatoid Arthritis (WPAI-RA) instrument was used to measure overall work productivity and impairment of regular activities during the past 7 days. Scores are calculated as impairment percentages [28], with higher percentages indicating greater work and activity impairment and less productivity.

The PROs were assessed at baseline, week 1, week 2, week 4, and every 4 weeks thereafter to week 24; after week 24 they were assessed at weeks 32, 40, and 52; with the exception of the FACIT-F, which was assessed at baseline, week 1, week 4 and then followed the same schedule as the other PROs and the SF-36, EQ-5D, and WPAI-RA, which were assessed at baseline and week 4 and then followed the same schedule as the other PROs.

### Statistical analysis

Randomized patients were included in the analyses under a modified intention-to-treat principle (mITT analysis set), which included all patients treated with ≥ 1 dose of study drug. Treatment comparisons for categorical and continuous measures were performed using logistic regression and analysis of covariance (ANCOVA), respectively, with baseline value (for continuous measures only), treatment, geographical region, and presence of baseline joint erosions (yes/no) in the model. The Fisher exact test was used for categorical data when sample size requirements for the logistic regression model were not met (< 5 responders in any category for any factor). Differences in the duration of MJS were assessed with the Wilcoxon rank sum test. Any duration of MJS lasting > 12 hours was truncated to 720 minutes for the purpose of this analysis.

Patients who were rescued or discontinued from the study or study treatment were thereafter defined as non-responders (non-responder imputation (NRI)) for all categorical data. These patients also had their last observations before rescue or discontinuation (modified last observation carried forward (mLOCF)) used for analyses of continuous data. The WPAI-RA analyses were censored after rescue or study discontinuation without imputation applied.

## Results

### Patients

A total of 588 patients were randomized and 584 patients received treatment; 210 initially received MTX, 159 baricitinib monotherapy, and 215 baricitinib + MTX. Patient demographics and disposition have been described by Fleischmann et al. [1]. Briefly, baseline demographics and clinical characteristics were similar among treatment groups (Table 1) [1]. The median disease duration was 0.2 years and more than 90% of patients were DMARD-naïve. The mean MTX dose achieved was 17.7 mg per week in both the MTX and combination groups; approximately 23% of patients received the lower MTX dose regimen. The mean doses prescribed in the full-dose/low-dose groups were 19.6 mg/12.1 mg in the MTX monotherapy group and 19.2 mg/12 mg in the baricitinib + MTX group, respectively, at week 24. Patients had active disease, impaired physical function, moderate levels of pain and tiredness/fatigue, a median duration of MJS of 60–90 minutes, and reduced HRQOL (Table 1) at baseline.

**Table 1:** Patient characteristics, disease activity, and patient-reported outcomes at baseline*

Parameter^a^	MTX (*N* = 210)	Baricitinib 4 mg (*N* = 159)	Baricitinib 4 mg + MTX (*N* = 215)
Age, years	50.5 (13)	50.9 (13)	48.5 (14)
Female, *n* (%)	148 (70)	121 (76)	156 (73)
Duration of RA, years	1.3 (4.0)	1.9 (4.7)	1.3 (2.7)
Duration of RA, years, median	0.2	0.2	0.2
Concomitant corticosteroid use, *n* (%)	76 (36)	47 (30)	83 (39)
Number of previous cDMARDs used, *n* (%)			
0	190 (90)	146 (92)	197 (92)
1	20 (10)	13 (8)	18 (8)
2	0	0	0
Ever used DMARD (≤ 3 doses of MTX permitted), *n* (%)	20 (10)	13 (8)	18 (8)
Disease activity			
Swollen joint count, of 66	16 (11)	16 (9)	16 (10)
Tender joint count, of 68	27 (15)	26 (14)	28 (15)
DAS28-hsCRP	5.9 (1.0)	5.9 (1.0)	5.9 (0.9)
DAS28-ESR	6.6 (1.0)	6.6 (1.1)	6.6 (1.0)
Simplified Disease Activity Index	42 (14)	43 (14)	43 (13)
Clinical Disease Activity Index	39 (13)	40 (13)	40 (13)
Patient-reported outcomes			
HAQ-DI (0–3)	1.7 (0.7)	1.6 (0.7)	1.6 (0.7)
PtGA VAS (0–100)	66 (24)	65 (22)	63 (24)
Patient’s assessment of pain, VAS (0–100)	65 (24)	64 (22)	63 (23)
Fatigue (FACIT-F; 0–52)	27 (11)	28 (11)	28 (11)
Median (IQR) duration of morning joint stiffness, minutes	90 (30, 180)	60 (30, 180)	90 (30, 180)
Worst Joint Pain NRS (0–10)	7 (2)	7 (2)	7 (2)
Worst Tiredness NRS (0–10)	6 (2)	6 (3)	6 (2)
QoL (SF-36; 0–100)			
PCS	32 (8)	33 (8)	32 (9)
MCS	47 (12)	45 (13)	47 (13)
EQ-5D			
Health State Index Score, UK algorithm (-0.594,1)	0.473 (0.256)	0.485 (0.255)	0.489 (0.251)
Self-perceived health score VAS (0–100)	51 (23)	50 (23)	51 (23)

Score ranges for individual patient-reported outcomes measures are indicated in brackets. Lower scores indicate better outcomes for HAQ-DI, PtGA, patient’s assessment of pain, Worst Joint Pain, Worst Tiredness. Higher scores indicate better outcomes for FACIT-F, SF-36 and EQ-5D

*EQ-5D* European Quality of Life-5 Dimensions, *FACIT-F* Functional Assessment of Chronic Illness Therapy–Fatigue, *HAQ-DI* Health Assessment Questionnaire-Disability Index, *IQR* interquartile range, *MCS* Mental Component Score, *MTX* methotrexate, *NRS* numeric rating scale, *PCS* Physical Component Score, *PtGA* Patient’s Global Assessment of Disease Activity, *QoL* quality of life, *RA* rheumatoid arthritis, *SF-36* Short Form-36, *UK* United Kingdom, *VAS* visual analog scale

^a^Data are presented as mean (standard deviation) unless stated otherwise

^*^Further details can be found in Fleischmann et al. 2017 [1]

### PROs

In the RA-BEGIN study, although MTX monotherapy was an effective therapy in many patients, statistically significant improvements in PROs were seen at week 24 across the majority of measures and were maintained through week 52 for both the baricitinib monotherapy and baricitinib + MTX groups compared to MTX. For some measures, statistically significant improvements were seen as early as week 1.

#### HAQ-DI, PtGA, and patient’s assessment of pain

As reported by Fleischmann et al. [1] for HAQ-DI, PtGA and patient’s assessment of pain, improvements in both the baricitinib monotherapy and baricitinib + MTX groups were evident as early as week 1 compared to MTX. Significant improvements in physical function, PtGA and patient’s assessment of pain were maintained through week 24 to week 52 (Table 2).

**Table 2:** Change from baseline at week 24 and week 52 for patient-reported outcomes

PRO measure^a^ (95% CI)		Week 24			Week 52	
	MTX (*N* = 210)	Baricitinib 4 mg (*N* = 159)	Baricitinib 4 mg + MTX (*N* = 215)	MTX (*N* = 210)	Baricitinib 4 mg (*N* = 159)	Baricitinib 4 mg + MTX (*N* = 215)
Physical Function (HAQ-DI)	− 0.74 (− 0.83, − 0.66)	− 1.04 (− 1.14, − 0.95)***	− 1.03 (− 1.11, − 0.95)***	− 0.71 (− 0.79, − 0.62)	− 0.99 (− 1.08, − 0.89)***	− 1.06 (− 1.14, − 0.97)***
Patient’s Global Assessment of Disease Activity (PtGA)	− 31 (− 34, − 27)	− 41 (− 45, − 38)***	− 40 (− 43, − 37)***	− 29 (− 32, − 26)	− 40 (− 44, − 37)***	− 43 (− 46, − 39)***
Patient’s assessment of pain	− 30 (− 33, − 27)	− 41 (− 45 − 37)***	− 41 (− 44, − 38)***	− 31 (− 34, − 27)	− 40 (− 44, − 37)***	− 43 (− 47, − 40)***
FACIT-F	8.9 (7.6, 10.1)	13.3 (11.8, 14.7)***	12.2 (11.0, 13.5)***	8.9 (7.6, 10.2)	11.7 (10.2, 13.1)**	12.6 (11.4, 13.9)***
MJS Duration, median change from baseline	− 35.0 (− 55.0, − 25.0)	− 50.0 (− 60.0, − 30.0)	− 59.0 (− 85.0, − 40.0)**	− 40.0 (− 55.0, − 30.0)	− 55.0 (− 60.0, − 40.0)	− 60.0 (− 80.0, − 50.0)**
Worst Joint Pain NRS	− 2.8 (− 3.2, − 2.5)	− 4.0 (− 4.3, − 3.6) ***	− 4.0 (− 4.3, − 3.7)***	− 3.0 (− 3.4, − 2.7)	− 3.9 (− 4.3, − 3.6)***	− 4.1 (− 4.4, − 3.8)***
Worst Tiredness NRS	− 2.1 (− 2.5, − 1.8)	− 3.1 (− 3.5, − 2.7)***	− 3.0 (− 3.3, − 2.7)***	− 2.2 (− 2.5, − 1.8)	− 3.0 (− 3.4, − 2.6)**	− 2.9 (− 3.3, − 2.6)**
EuroQol-5 Dimensions (EQ-5D)						
Health State Index Score, UK algorithm	0.200 (0.173, 0.227)	0.288 (0.257, 0.318)***	0.288 (0.261, 0.315)***	0.191 (0.161, 0.221)	0.272 (0.239, 0.306)***	0.273 (0.244, 0.303)***
US algorithm	0.138 (0.119, 0.158)	0.199 (0.177, 0.221) ***	0.199 (0.179, 0.218) ***	0.133 (0.111, 0.154)	0.187 (0.163, 0.211)***	0.189 (0.168, 0.210)***
Self-perceived health score	15 (12, 18)	24 (20, 27)***	22 (18, 25)**	14 (11, 18)	25 (21, 28)***	25 (22, 28)***

*CI* confidence interval, *MJS* morning joint stiffness, *MTX* methotrexate, *NRS* numeric rating scale, *PRO* patient-reported outcomes, *RA* rheumatoid arthritis

^a^Data are presented as least-squares mean unless stated otherwise

*p ≤ 0.05; **p ≤ 0.01; ***p ≤ 0.001 vs. MTX

The percentages of patients with improvement in HAQ-DI scores that exceeded the MCID (≥ 0.22) with MTX, baricitinib monotherapy, and baricitinib + MTX, respectively, were 70%, 81%, and 79% (*p* ≤ 0.05 for both baricitinib groups vs. MTX) at week 24 and were 57%, 68%, and 72% (*p* ≤ 0.05 for baricitinib monotherapy vs. MTX; *p* ≤ 0.001 for baricitinib + MTX vs. MTX) at week 52. The percentage of patients who achieved the normative value of ≤ 0.25 at week 24 for MTX, baricitinib monotherapy, and baricitinib + MTX, respectively, were 21%, 33%, and 34% (*p* ≤ 0.05 for the baricitinib monotherapy vs. MTX; *p* ≤ 0.01 for baricitinib + MTX vs. MTX) and were 18%, 29%, and 34% (*p* ≤ 0.05 for the baricitinib monotherapy vs. MTX; *p* ≤ 0.001 for baricitinib + MTX vs. MTX) at week 52.

#### FACIT-F, duration of MJS, worst joint pain and worst tiredness

**Fig. 1:**
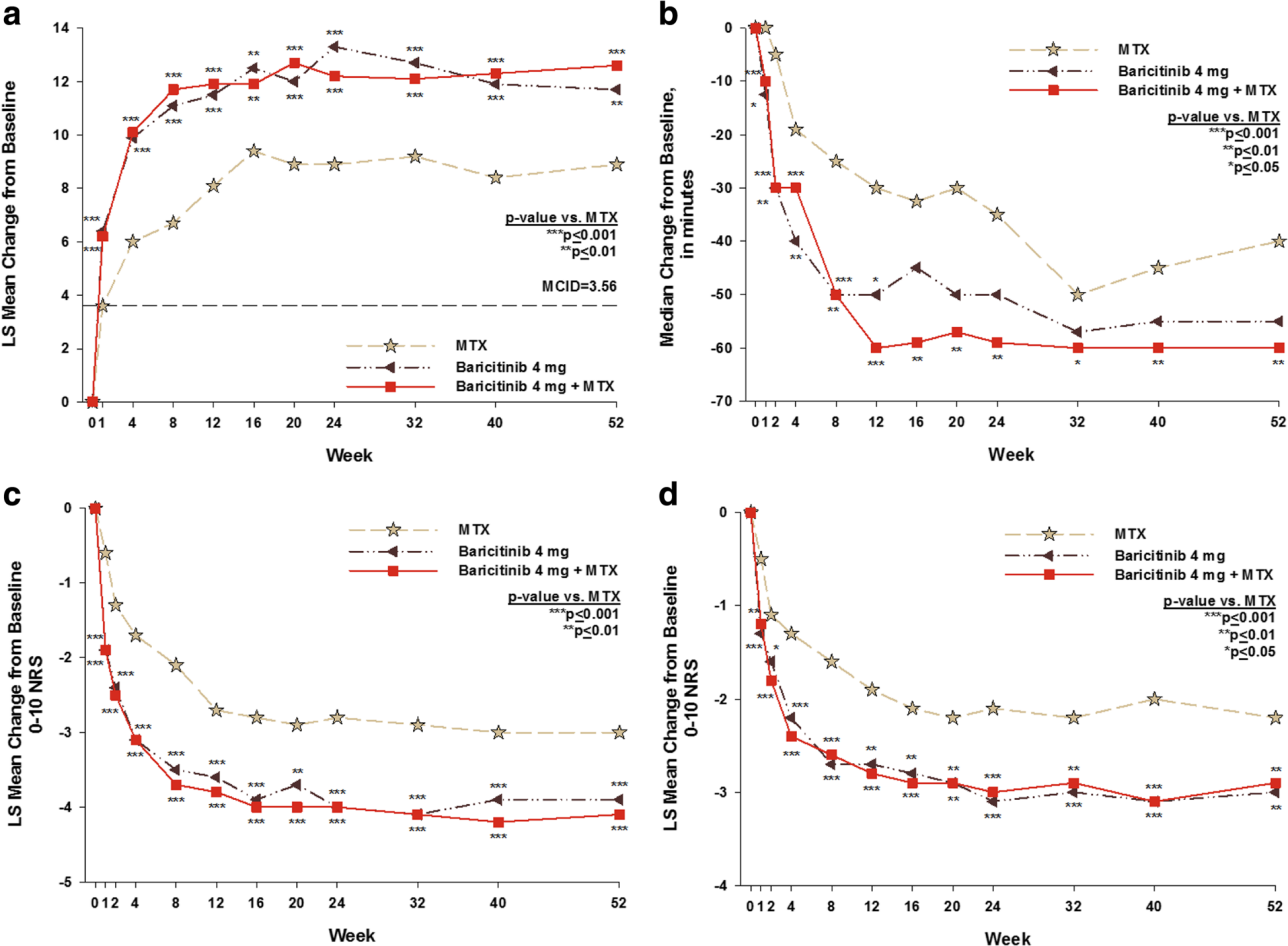
Change from baseline over time for the Functional Assessment of Chronic Illness Therapy-Fatigue (FACIT-F) (**a**), morning joint stiffness duration (**b**), worst joint pain (**c**), and worst tiredness (**d**). The dashed line represents the minimum clinically important difference (MCID) in the FACIT-F (≥ 3.56 points). The question asked for morning joint stiffness duration was “Please indicate how long your morning joint stiffness lasted yesterday (duration in minutes).” The question for worst joint pain was “Please rate your joint pain by selecting the one number that describes your joint pain at its WORST in the last 24 hours (0 = no pain; 10 = pain as bad as you can imagine).” The question for worst tiredness was “Please rate your tiredness by selecting the one number that describes your WORST level of tiredness during the past 24 hours (0 = no tiredness; 10 = as bad as you can imagine)”. MTX methotrexate

Compared to MTX monotherapy, statistically significant improvements in the FACIT-F for both baricitinib groups were observed as early as the first assessment at week 1 (*p* ≤ 0.001 for both baricitinib groups vs. MTX; Fig. 1). The duration of MJS, worst joint pain, and worst tiredness were significantly reduced in both baricitinib groups compared to MTX from week 1 (MJS duration, *p* ≤ 0.05 for baricitinib monotherapy vs. MTX, *p* ≤ 0.001 for baricitinib + MTX vs. MTX; worst joint pain, *p* ≤ 0.001 for both baricitinib groups vs. MTX; worst tiredness, *p* ≤ 0.001 for baricitinib monotherapy vs. MTX, *p* ≤ 0.01 for baricitinib + MTX vs. MTX; Fig. 1). The improvements in the FACIT-F score and reductions in duration of MJS, worst joint pain, and worst tiredness were maintained to week 24 and week 52 in both baricitinib groups (Fig. 1 and Table 2). The duration of MJS was not statistically significantly different, however, in the baricitinib monotherapy group vs. the MTX group at week 24 or week 52.

The percentage of patients with improvement in the FACIT-F that exceeded the MCID at week 24 (≥ 3.56) was 65%, 75%, and 71% for MTX, baricitinib monotherapy, and baricitinib + MTX, respectively, at week 24 (*p* ≤ 0.05 for baricitinib monotherapy vs. MTX; *p* = 0.268 for baricitinib + MTX vs. MTX). The percentage of patients with improvement in the FACIT-F that exceeded the MCID (≥ 3.56) was 54%, 62%, and 62%, respectively at week 52 (neither baricitinib group was statistically significantly different from the MTX group).

#### Health-related quality of life

**Fig. 2:**
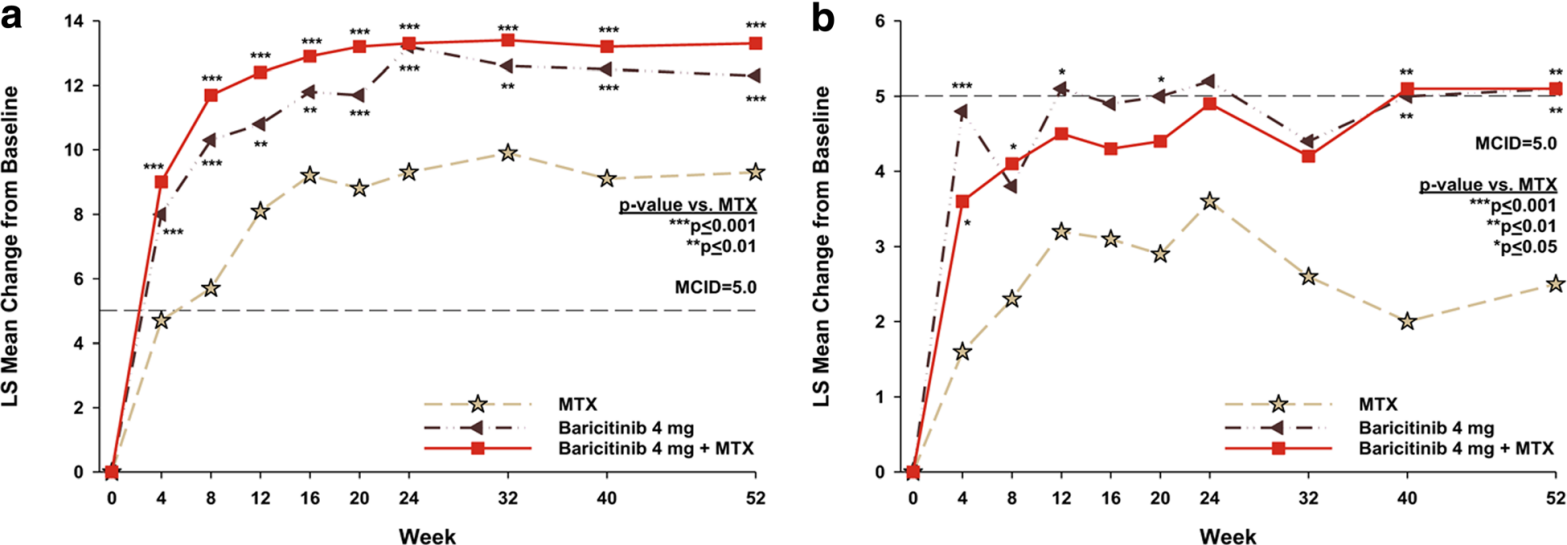
Change from baseline over time in the Physical Component Score (PCS) (**a**) and Mental Component Score (MCS) (**b**) of the Short Form-36 (SF-36). The dashed line represents the minimum clinically important difference (MCID) in the SF-36 PCS (≥ 5 points) and SF-36 MCS (≥ 5 points). MTX methotrexate

Compared to MTX monotherapy, statistically significant improvements in SF-36 PCS were observed in both the baricitinib monotherapy and the baricitinib + MTX groups as early as the first assessment at week 4 (Fig. 2); these improvements were maintained during the study. For the SF-36 MCS, numeric improvements were observed at all time points in both baricitinib treatment groups compared to the MTX group and the differences were statistically significant at weeks 4, 40 and 52 (*p* ≤ 0.05).

For the SF-36 PCS, at week 24, the percentage of patients who met or exceeded the MCID for MTX, baricitinib monotherapy, and baricitinib + MTX, respectively, was 62%, 71%, and 74% (*p* = 0.091 for baricitinib monotherapy vs. MTX; *p* ≤ 0.05 for baricitinib + MTX vs. MTX). At week 52, the percentage of patients who met or exceeded the MCID for the SF-36 PCS was 48%, 65%, and 65%, respectively (*p* ≤ 0.05 for baricitinib monotherapy vs. MTX; *p* ≤ 0.001 for baricitinib + MTX vs. MTX). In contrast, for the SF-36 MCS, there were no statistically significant differences between the baricitinib groups and the MTX group in the percentage of patients who met or exceeded the MCID.

For the EQ-5D UK index score at week 4, the first post-baseline assessment, there were statistically significant improvements in both the baricitinib monotherapy and baricitinib + MTX groups compared to MTX (*p* ≤ 0.001 for both baricitinib groups vs. MTX). These differences were maintained through week 52 (Table 2). A statistically significant improvement was seen in the EQ-5D VAS in both baricitinib groups compared to the MTX group, beginning at week 4 (*p* ≤ 0.001 for both baricitinib groups vs. MTX). These results were maintained through the end of the study (Table 2). Similar results were seen for the US index score (Table 2).

#### WPAI-RA

For the WPAI-RA assessment, patients in the baricitinib monotherapy and baricitinib + MTX groups reported improved daily activity compared to MTX monotherapy (Table 3). Among those patients employed at baseline and who maintained employment at week 24, there were statistically significant improvements in both baricitinib groups compared to the MTX group across all measures; these statistically significant improvements were only maintained at week 52 in the baricitinib + MTX group compared to the MTX group, with respect to work productivity loss (*p* ≤ 0.05) (Table 4).

**Table 3:** Work Productivity and Activity Impairment Questionnaire - Rheumatoid Arthritis (WPAI-RA): impairment of regular activities among all patients at baseline and least-squares mean change from baseline at week 24 and at week 52

WPAI-RA question administered to all patients^a^									
	Baseline, Mean (SD)			Week 24, LSM (95% CI)			Week 52, LSM (95% CI)		
	MTX (*N* = 210)	Baricitinib 4 mg (*N* = 159)	Baricitinib 4 mg + MTX (*N* = 215)	MTX (*N* = 210)	Baricitinib 4 mg (*N* = 159)	Baricitinib 4 mg + MTX (*N* = 215)	MTX (*N* = 210)	Baricitinib 4 mg (*N* = 159)	Baricitinib 4 mg + MTX (*N* = 215)
Percent activity impairment due to RA	61 (26)	62 (25)	59 (25)	− 25 (− 29, − 22)	− 37 (− 40, − 33)***	− 33 (− 37, − 30)***	− 29 (− 32, −25)	− 35 (− 38, − 31)*	− 38 (− 42, − 35)***

*CI* confidence interval, *LSM* least-squares mean, *MTX* methotrexate, *RA* rheumatoid arthritis

^a^Data are presented as mean (standard deviation) unless stated otherwise

*p ≤ 0.05; ***p ≤ 0.001 vs. MTX

**Table 4:** Work Productivity and Activity Impairment Questionnaire - Rheumatoid Arthritis (WPAI-RA): mean presenteeism, absenteeism, and work productivity loss at baseline and least-squares mean change from baseline at week 24 and at week 52 among patients employed at baseline and at Week 24 or Week 52

WPAI-RA questions administered to patients who were employed^a^									
	Baseline, Mean (SD)			Week 24, LSM (95% CI)			Week 52, LSM (95% CI)		
	MTX (*N* = 210)	Baricitinib 4 mg (*N* = 159)	Baricitinib 4 mg + MTX (*N* = 215)	MTX (*n* = 85)	Baricitinib 4 mg (*n* = 63)	Baricitinib 4 mg + MTX (*n* = 105)	MTX (*n* = 67)	Baricitinib 4 mg (*n* = 58)	Baricitinib 4 mg + MTX (*n* = 96)
Percent employed at time point^b^, *n* (%)	94 (45)	67 (42)	117 (55)	80 (94)	59 (94)	94 (90)	57 (85)	55 (95)	80 (83)
Percent impairment while working due to RA (presenteeism)	49 (28)	49 (26)	50 (26)	− 20 (− 25, − 15)	− 29 (− 35, − 24)*	− 31 (− 35, − 26)**	− 26 (− 31, − 20)	− 29 (− 35, − 23)	− 31 (− 35, − 27)
Percent overall work impairment due to RA (work productivity loss)	53 (29)	51 (27)	55 (27)	− 18 (− 24, − 13)	− 29 (− 36, − 23)*	− 30 (− 35, − 25)**	− 23 (− 30, − 17)	− 30 (− 37 − 23)	− 32 (− 38, − 27)*
Percent work time missed due to RA (absenteeism)	19 (31)	14 (29)	15 (25)	− 2 (− 7, 2)	− 10 (− 15, − 5)*	− 9 (− 12, − 5)*	− 3 (− 7, 1)	− 8 (− 12, − 3)	− 8 (− 11, − 4)

*CI* confidence interval, *LSM* least-squares mean, *MTX* methotrexate, *RA* rheumatoid arthritis

^a^Data are presented as mean (standard deviation) or LSM (95% CI)

^b^For weeks 24 and 52, the percentage of patients employed at baseline and who continued to be employed at that time point

*p ≤ 0.05; **p ≤ 0.01 vs. MTX

## Discussion

The primary objective of the RA-BEGIN study was to evaluate baricitinib, an oral tsDMARD that selectively inhibits JAK1 and JAK2, as monotherapy or combined with MTX compared to MTX monotherapy in patients with active RA and no prior DMARD therapy or limited prior MTX therapy. The current analysis has focused on PROs which, in conjunction with clinical assessment measures, are a valuable and practical tool for comprehensive disease management and provide important insights into the patient’s response to therapy and as such, should be utilized in the clinic [29]. Although MTX was effective in this population, particularly at later time points, the majority of the pre-specified PROs of physical function, HRQOL, PtGA, pain, fatigue, duration of MJS, tiredness, and joint pain were all statistically significantly improved to a greater extent at many or all time points in patients treated with baricitinib monotherapy and baricitinib + MTX compared to the MTX monotherapy.

The clinical relevance of these PRO improvements with baricitinib is emphasized by their consistent superiority to MTX, the oral standard of care in the treatment of RA, in contrast to simply being compared to a placebo control. The ACR guidelines and EULAR recommendations propose that MTX be the first-line DMARD treatment for DMARD-naïve patients because of its effectiveness in controlling disease activity, improving patient function and limiting radiographic progression in up to one third of patients, and having an acceptable and well-known safety profile and a low cost [1, 6, 7]. These recommendations have relied on studies in MTX-naïve patients that compared MTX to bDMARD monotherapy and indicated that MTX was as clinically effective (including PRO data) as bDMARD monotherapy [30–32]. One study, TEMPO, used a similar design to RA-BEGIN to compare etanercept either as monotherapy or in combination with MTX vs. MTX in patients with mean disease duration of over 6 years. In TEMPO, etanercept monotherapy had similar benefit to MTX monotherapy in improving PROs [33].

There are, however, a number of patients for whom MTX monotherapy is unsuitable. There is, therefore, an unmet need for alternative therapeutic choices for such patients; novel, orally administered tsDMARDs, such as baricitinib, would appear to have desirable attributes in this setting. Importantly, in RA-BEGIN, the comparable effects on PRO improvements in the baricitinib monotherapy and baricitinib + MTX groups suggest that baricitinib may be an effective monotherapy treatment.

These results from the current analysis are similar to what has been reported from studies of bDMARDs plus MTX in patients with early RA. For example, the PREMIER trial compared MTX monotherapy, adalimumab monotherapy, and adalimumab + MTX in patients with early RA who were naive to MTX therapy [10]. For MTX, adalimumab, and adalimumab + MTX, respectively, the baseline HAQ-DI values were 1.5, 1.6, and 1.5 with decrements of − 0.8, − 0.8 and − 1.1 at 1 year [10]. In the current RA-BEGIN analysis, at baseline, the mean baseline HAQ-DI scores for MTX, baricitinib monotherapy, and baricitinib + MTX, respectively, were 1.7, 1.6, and 1.6 with decrements of − 0.71, − 0.99 and − 1.06 at one year. Similar trends were also observed in the ORAL Start trial, which compared tofacitinib monotherapy, both 5 and 10 mg twice daily, to MTX monotherapy in MTX-naïve patients. On physical functioning, tofacitinib 5 mg and 10 mg demonstrated benefit compared to MTX as monotherapy [11].

In RA-BEGIN, trends in the PRO results were also generally similar to PRO results from other studies. The HAQ-DI results in the OPTIMA trial were similar to the HAQ-DI observations described above [34]. In the OPTIMA trial, which compared adalimumab + MTX with MTX monotherapy in patients with RA who were naïve to MTX therapy, the baseline mean HAQ-DI values were 1.61 for adalimumab + MTX and 1.60 for MTX. At week 26, the mean HAQ-DI values were 0.7 for adalimumab + MTX and 0.9 for MTX (p < 0.001) [34].

Similar trends were observed with the FACIT-F, which measures fatigue, a major concern for patients and an important outcome measure in RA studies [35]. Notably, as with most other PROs, it is not captured in the ACR response criteria or Disease Activity Score (DAS) response composite indices. The FACIT-F was assessed in the ORAL Start trial. At 6 months, the least-squares mean (LSM) changes from baseline for MTX, tofacitinib 5 mg, and tofacitinib 10 mg, respectively, were 6.3, 8.7, and 9.1 [11, 36]. In the current analysis, the LSM changes from baseline to 6 months for MTX, baricitinib monotherapy, and baricitinib + MTX were 8.9, 13.3, and 12.2.

For the HRQOL assessments, patients in both baricitinib treatment groups reported statistically significant improvements in the EQ-5D index scores and VAS scores and the SF-36 PCS measure compared with MTX monotherapy at 4 weeks post baseline. These results were maintained through week 52. For the SF-36 MCS measure there was no statistically significant difference in the percentage of patients who met or exceeded the MCID (≥ 5) across the treatment groups. These SF-36 MCS results are consistent with previous observations from other clinical trials [19, 20]. Of note, the baseline values for the SF-36 MCS ranged from 45 to 47 in the current analysis, which demonstrates a modest, rather than severe impairment. Patients, therefore, may have had less opportunity to improve their SF-36 MCS scores.

Work productivity and activity impairment have a significant economic impact on patients. In this trial, all three treatments were effective in improving work impairment, with earlier benefit noted in both baricitinib groups, but with comparable benefits at one year.

This study has a number of limitations. In clinical practice, in contradistinction to this study, MTX will typically be started either as monotherapy or in combination with other csDMARDs prior to the use of a bDMARD or a tsDMARD, such as baricitinib. However, the intent of this study was to compare the effectiveness of baricitinib to MTX and given current treatment paradigms, this is best accomplished in MTX-naïve patients. Following initial escalation, the maintenance dose of MTX was limited to no more than 20 mg/week; it is conceivable that there could have been a better response with MTX if the dose could have been increased or switched to subcutaneous MTX. In addition, other initial treatment regimens, such as MTX in combination with other csDMARDs, were not evaluated. Additionally, the use of carrying forward the last observations before rescue or discontinuation assumes that the PRO values would not change over time had these events not occurred. This is an appropriate statistical method for this analysis, but the assumption is not verifiable. Last, as in most double-blind comparator trials, the inclusion and exclusion criteria limited the participation of some patients, who are routinely seen in clinical practice. This may potentially impact the generalizability of the study results.

## Conclusions

We have shown that the majority of the pre-specified PRO measures in this study improved in each of the active treatment groups evaluated, but there were statistically and clinically significant improvements with either baricitinib group over MTX monotherapy, many from as early as week 1. Early improvement in PROs is an important clinical outcome. The PRO data from this trial reinforce the outcomes for other signs and symptoms observed in the RA-BEGIN study [1]. This trial demonstrates that baricitinib may either be used as monotherapy or in combination with MTX, as improvements across measures of how patients feel and function were comparable for each regimen, both appearing consistently more effective than MTX. This may be of importance to physicians and their patients for whom use of MTX is not desirable, and adds to previously published data showing that baricitinib can be an effective agent in the treatment of RA [1, 37].

## Abbreviations

ACR: American College of Rheumatology
ANCOVA: Analysis of covariance
csDMARDs: Conventional synthetic disease-modifying anti-rheumatic drugs
DAS: Disease Activity Score
EQ-5D: EuroQol-5 Dimensions
EULAR: European League Against Rheumatism
FACIT-F: Functional Assessment of Chronic Illness Therapy-Fatigue
HAQ-DI: Health Assessment Questionnaire-Disability Index
HRQOL: Health-related quality of life
JAK: Janus kinase
MCID: Minimum clinically important difference
mITT: Modified intention-to-treat
MJS: Morning joint stiffness
mLOCF: Modified last observation carried forward
MTX: Methotrexate
NRI: Non-responder imputation
NRS: Numeric rating scale
OMERACT: Outcome Measures in Rheumatology
PROs: Patient-reported outcomes
PtGA: Patient’s Global Assessment of Disease Activity
QD: Twice daily
RA: Rheumatoid arthritis
SF-36 MCS: Short Form-36 Mental Component Score
SF-36 PCS: Short Form-36 Physical Component Score
tsDMARDs: Targeted synthetic DMARDs
VAS: Visual analog scale
WPAI-RA: Work Productivity and Activity Impairment-Rheumatoid Arthritis
